# Approaches to canine health surveillance

**DOI:** 10.1186/2052-6687-1-2

**Published:** 2014-04-16

**Authors:** Dan G O’Neill, David B Church, Paul D McGreevy, Peter C Thomson, Dave C Brodbelt

**Affiliations:** Veterinary Epidemiology, Economics and Public Health, The Royal Veterinary College, Hawkshead Lane, North Mymms, Hatfield, Herts, AL9 7T UK; Small Animal Medicine and Surgery Group, The Royal Veterinary College, Hawkshead Lane, North Mymms, Hatfield, Herts, AL9 7TA UK; Faculty of Veterinary Science, The University of Sydney, R.M.C. Gunn Building (B19), Sydney, NSW 2006 Australia

**Keywords:** Surveillance, Epidemiology, Canine, Data source, Primary-care practice, Referral practice, Insurance, Questionnaire, Health scheme, Cancer registry, Disorder

## Abstract

Effective canine health surveillance systems can be used to monitor disease in the general population, prioritise disorders for strategic control and focus clinical research, and to evaluate the success of these measures. The key attributes for optimal data collection systems that support canine disease surveillance are representativeness of the general population, validity of disorder data and sustainability. Limitations in these areas present as selection bias, misclassification bias and discontinuation of the system respectively. Canine health data sources are reviewed to identify their strengths and weaknesses for supporting effective canine health surveillance. Insurance data benefit from large and well-defined denominator populations but are limited by selection bias relating to the clinical events claimed and animals covered. Veterinary referral clinical data offer good reliability for diagnoses but are limited by referral bias for the disorders and animals included. Primary-care practice data have the advantage of excellent representation of the general dog population and recording at the point of care by veterinary professionals but may encounter misclassification problems and technical difficulties related to management and analysis of large datasets. Questionnaire surveys offer speed and low cost but may suffer from low response rates, poor data validation, recall bias and ill-defined denominator population information. Canine health scheme data benefit from well-characterised disorder and animal data but reflect selection bias during the voluntary submissions process. Formal UK passive surveillance systems are limited by chronic under-reporting and selection bias. It is concluded that active collection systems using secondary health data provide the optimal resource for canine health surveillance.

## Lay summary

An ability to identify at a population level how many dogs, within breeds or across all breeds develop certain diseases, either over a fixed time period (e.g. each year) or as a proportion of the total population is very important. This helps establish whether some animals and breeds are particularly susceptible to a disease or whether conditions are becoming more or less common. The percentage of affected dogs in a population is called the ***disease prevalence*** and the number of new cases of disease in a year is called the ***disease incidence***. These are critical measurements for studying patterns of health and disease and form a branch of medicine called ***epidemiology***. Veterinary epidemiology has been slow to develop but is now recognised as being critical for improving canine health and welfare. To do this effectively, information and health records about dogs, have to be collected. This should be done in a standardised way where the same terms are used to describe diseases and symptoms and laboratory tests. The collection of disease information is described as being ***health surveillance*** or ***disease monitoring***. To be effective, systems for doing this should be representative of the whole population. Methods for doing this are now developing but until recently the only ways to collect such information were by using records from pet insurance companies or from veterinary referral clinics. Problems can exist with such data as they can have a **selection or referral bias** and not truly represent the picture at the population level or include information that is incorrect due to ***disease misclassification***. This review describes the history and development of health surveillance systems in canine medicine and what their strengths and weaknesses are. It also describes some of the new ways this is now being taken forward to collect high quality health data to support clinical and genetic studies.

## Introduction

Disease surveillance describes the monitoring of population health to ascertain the existence and changes in disease levels in combination with an appropriate mitigation plan once disease levels become inordinate [1–3] and is now an established veterinary activity for disease control [4]. Effective canine health surveillance provides information that supports disorder prioritisation, improved disorder management, focussed clinical research, advice on breed standards reform and regulation to improve animal welfare [5]. Optimal data sources for canine disease surveillance require representativeness of the general population, a well-defined denominator population, validity of disorder diagnosis data and sustainability. Limitations in these areas present as selection bias, misclassification bias and discontinuation of the system respectively [4].

Amongst other epidemiological applications, health surveillance data can be analysed to derive disorder prevalence proportion (proportion of animals affected) and incidence risk (proportion of previously healthy animals that become diseased over a specified period) estimates, perform risk factor (attributes associated with disease occurence) studies and examine survival in affected dogs [1]. Prevalence data are currently available on only 1% of inherited disorders affecting popular UK dog breeds [6]. A deficiency of disorder prevalence information relating to UK dogs has been identified as a major constraint to effective reforms to purebred dog health [7–9]. Novel epidemiological information on disorders in dogs can assist with welfare prioritisation of disorders for appropriate focus of research efforts and breeding programs [6, 10]. Increased awareness by veterinarians of disorder frequency and survival can improve diagnostic protocols, optimise case management and enhance prognostic advice given to clients [11].

Collection processes for surveillance data may be passive or active [4]. Passive collection occurs at the discretion of the owner or veterinarian, whose willingness or ability to participate can limit the validity of the emergent data [12]. Passive systems typically suffer from incomplete reporting, selection bias and frequently lack a defined denominator population [13]. Active collection describes systematic data collection methods, usually from a defined population, location and timespan, and can be relatively timely, complete and accurate [13].

Data used to support health surveillance may be primary or secondary. Primary data are collected specifically for the research while secondary data have been pre-collected by a third party for some other reason. Primary data collection offers better control of the types and quality of the data and may be easier to validate. However, secondary data sources may offer more efficient collection, reduced costs, larger sample size, better representativeness and reduced bias [14]. Secondary sources of health data for dogs include pet insurance, referral practice and primary-care practice records [14–16].

Surveillance data may be formatted as unstructured (free-form text), semi-structured (non-standardised lists) or structured data (standardised coding) [17]. Unstructured data are problematic for large-scale studies because of colloquial language, non-standard abbreviations and misspellings [18, 19] but may provide contextual information that is otherwise unavailable [20]. Analytic methods for free-form text are progressing [21]. Structured data entry using standardised coding terminologies including the VeNom codes [22] and the AAHA (American Animal Hospital Association) Diagnostic Terms [23] may improve analytic efficiency [24, 25].

Obstacles to effective data collection, analysis and interpretation are common to both human and animal surveillance. These include ethical constraints [26], data warehousing [27], setting valid case definitions [28], data quality and missing data [29, 30], appropriate coding systems [31], bias [32], generalisability [33], participation [34] linking data sources [35], financial cost [36], clinical coding [37], developing syndromic surveillance [38] and impact assessment [39]. Veterinary surveillance methods can benefit from experiences gained during the development of human methods [40]. Further opportunities for improved veterinary surveillance methods stem from the advent of Big Data techniques for data management, analysis and accessibility [41], and developments in the science of natural language processing (NLP) [42, 43].

Diverse data sources have been used to support canine disorder surveillance but there is an absence of a universally-accepted standard for ‘good practice’ in veterinary surveillance methodology [44]. This review is intended to evaluate current sources of canine health data and to identify their strengths and weaknesses as surveillance sources in order to assist with interpretation of results from studies based on these data.

## Review

### Pet insurance databases

Animal insurance databases have been increasingly used for epidemiological research since the 1970s [45]. A literature review in 2009 of publications based on dog insurance data identified 16 Swedish studies using Agria Insurance data (http://www.agriavet.co.uk/) and three UK studies using PetProtect Insurance data (http://www.petprotect.co.uk/) [15]. About 20 pet insurance providers [46] insure an estimated 34.0-40.3% of UK dogs [46, 47]. In Sweden, 68.4% of dogs are insured, with 61.0% of these insured dogs being covered by Agria Pet Insurance [48] alone [49]. Estimates suggest that just 4% of dogs in Canada [50] and 0.3-3.0% of dogs in America [51, 52] are insured.

For research purposes, insurance databases benefit from holding information on both the numerator clinical events and the denominator insured animals across large populations of dogs [15]. Validation studies between Agria Insurance demographic data and veterinary clinical records showed high agreement for dog breed (95%) and sex (99%), fair agreement for diagnosis (84%) but only moderate agreement for year of birth (66%), suggesting that insurance data are of adequate quality for research purposes [25]. Location information within insurance records allows spatial analysis of geographic risk factors. Post-code data have been used to show associations between the incidence of canine atopic dermatitis and average annual rainfall levels, proximity to a veterinary dermatologist, country sector and increased human population [53].

Although technically easy to analyse [25] and their large size lending statistical power to gain meaningful results even for uncommon breeds [54, 55], insurance data have some important limitations. Diagnostic term validity may vary between disorders depending on the ease of clinical diagnosis, the veterinarian’s clinical acumen and the veterinary practice’s facilities. For example, diagnosis validation for atopic dermatitis claims showed high agreement with veterinary medical records that claim dogs had allergic skin disease (97.6%) but only moderate agreement for full atopic dermatitis diagnostic criteria (40.9-84.2%) [15, 56].

Insured dogs may poorly represent the wider national dog population [15]. Insurance coverage varies with breed and purebred status [49] and life-cover may end when dogs reach 10 years of age [57]. Younger animals were heavily overrepresented in a UK insured population [58], requiring age-standardisation of results for generalisation [59]. Insured animals may receive more-frequent veterinary visits and undergo more medical procedures than non-insured animals [15]. A UK study using primary-care practice electronic patient record (EPR) data showed that insured dogs had over twice the odds of a diagnosis of chronic kidney disease compared with uninsured dogs [11]. Insurance status may even affect mortality by impacting on euthanasia decisions [15].

Insurance data include only clinical events that are non-excluded and where the cost exceeds the deductible excess. Exclusions have tended to increase over time and vary by policy, breed and the medical history of individual animals [15, 60]. Insurance claim levels may also vary between breeds. For example, death claims with an associated diagnosis were received for just 50% of insured crossbred dogs compared with over 80% of insured Bernese Mountain Dogs and Cavalier King Charles Spaniels [15]. Cohort insurance studies to monitor health status within individual animals over time can be problematic because repeat-disorder claim levels are affected by changing insurance status and dynamic exclusions applied over time based on claim history [61].

Insurance data research has lead to many useful publications on dogs covering specific disorders and overall morbidity and mortality (Table 1). Good understanding and interpretation of limiting factors are important when considering insurance data for canine health surveillance. Proposed studies should be considered on an individual basis or possibly even abandoned in the case of unavailable or inaccurate data [15]. Swedish insurance studies have benefited from an open approach to data sharing and strong research collaboration between Agria Insurance and academic colleagues that could be mirrored in other countries [55].

**Table 1:** **Selected published and findings studies on dog health based on insurance data**

Topic & main conclusion	Insurer	Country	Reference
German Shepherd Dog, predisposed to immune-mediated diseases	Agria^a^	Sweden	Vilson *et al.* [62]
Atopic dermatitis, offspring of bitches fed non-commercial diet during lactation protected	Agria^a^	Sweden	Nodtvedt *et al.* (2007) [53]
Bone tumours, Irish Wolfhound, St. Bernard, and Leonberger at increased risk	Agria^a^	Sweden	Egenvall *et al.* (2007) [63]
Cancer, skin and soft tissue tumours had highest prevalence	PetProtect^b^	UK	Dobson *et al.* (2002) [58]
Demography, insured dogs similar to the general dog population	Agria^a^	Sweden	Sallander (2001) [64]
Diabetes mellitus, highest incidence in Australian Terrier, Samoyed, Swedish Elkhound and Swedish Lapphund	Agria^a^	Sweden	Fall *et al.* (2007) [65]
Dystocia/caesarean section, Scottish terrier at increased risk	Agria^a^	Sweden	Bergstrom (2006) [66]
Heart disease, Irish Wolfhound, Cavalier King Charles Spaniel and Great Dane showed highest mortality	Agria^a^	Sweden	Egenvall *et al.* (2006) [67]
Intervertebral disc degeneration, Miniature Dachshund, Standard Dachshund and Doberman Pinscher had highest incidence	Agria^a^	Sweden	Bergknut *et al.* (2012) [68]
Lymphoma, Bull mastiff, Bulldog and Boxer had high incidence	PetProtect^b^	UK	Edwards *et al.* (2003) [69]
Mammary tumours, highest incidence in English Springer Spaniel, Doberman and Boxer	Agria^a^	Sweden	Egenvall *et al.* (2005b) [70]
Morbidity and mortality, marked breed differences in survival	Agria^a^	Sweden	Bonnett and Egenvall (2010) [55]
Morbidity and mortality (1995–1996), insurance data useful for epidemiological studies	Agria^a^	Sweden	Egenvall *et al.* (2000a) [60]
Mortality, wide breed differences in survival	Agria^a^	Sweden	Egenvall *et al.* (2000b) [71]
Mortality, >40% of deaths from trauma, tumours and locomotor disorders	Agria^a^	Sweden	Bonnett *et al.* (1997) [72]
Mortality (1995–2000), Irish Wolfhound and Great Dane had highest mortality	Agria^a^	Sweden	Egenvall *et al.* (2005a) [57]
Mortality (1995–2000), 62% of deaths from tumour, trauma, locomotor, heart and neurological disorders	Agria^a^	Sweden	Bonnett *et al.* (2005) [73]
Pyometra, increased risk Rough Collies, Rottweilers, Cavalier King Charles Spaniels, Golden Retrievers, Bernese Mountain Dogs, and English Cocker Spaniels	Agria^a^	Sweden	Egenvall *et al.* (2001) [74]

^a^ http://www.agriavet.co.uk ^b^ http://www.petprotect.co.uk.

### Referral practice clinical records

The Veterinary Medical Data Base (VMDB) holds 7 million standardised abstracted records from 26 veterinary schools in the US and Canada [75] with a coding system that records diagnostic terms using either pathophysiologic, histologic or descriptive terminologies [76]. VMDB data mining is based on discrete factors including breed, age, sex and diagnostic code and non-associated institutions are charged for data searches.

The large study population lends high statistical power to VMDB analyses, enabling exploration of rare disorders or disorder-within-breed studies [12] such as thyroid cancer, 0.2% prevalence [77], discospondylitis, 0.2%, [78], bronchiectasis, 0.05%, [79] and leptospirosis, 0.04% [80]. Other prevalence studies that used VMDB data have investigated cataract [81], glaucoma [81] and cardiac tumours [82]. However, VMDB studies are limited by inconsistencies in data completeness and quality, and by the mixing of referral data with some primary-care data [15]. Only nine of the contributing universities use the structured SNOMED coding system that links clinical care events to terms selected from a comprehensive list of disorder concepts and descriptions [83, 84]. The currency of the VMDB data is low, given that just 14 universities have uploaded data since the year 2000 [12, 75].

Referral data spanning 1995–2010 from the University of California-Davis Veterinary Medical Teaching Hospital were analysed to report purebred dog predispositions to 24 inherited disorders [85]. The authors considered that more intensive evaluation within breeds with published disorder predisposition and increased willingness of owners of purebred dogs to spend heavily on clinical investigations may lead to over-representation of some disorders in specific breeds and in purebreds more generally [86].

Despite the promise of good reliability for diagnoses from referral clinical data, referral biases towards complicated cases requiring more specialised care and towards locations closer to specialist centres limit the generalisability of study results [12]. Clients and animals that are referred are filtered by diagnostic work-ups, insurance status and financial considerations [15, 87]. Referral bias is likely to vary between disorders and to compromise the validity of prevalence studies that compare multiple disorders [12]. An ill-defined denominator population containing few healthy animals further limits referral data for prevalence estimation [88]. Referral clinical datasets may be less reliable for generalisable prevalence estimation and may be best reserved to test hypotheses relating to specific causal mechanisms [89].

### Primary-care practice clinical records

Analysis of primary-care practice data benefits from the cumulative clinical experience of general practitioners to offer unique insights into companion animal health [24] and can support an evidence-based approach that is relevant to primary-care practitioners [90].

An early example of primary-care practice surveillance (1998–2001) used manual paper-based data collection by veterinary students undergoing extramural studies to describe overall reasons for veterinary presentation and the prevalence of dermatological diagnoses [91]. This study concluded that, although practicable for short-term and highly focused studies, clinical research using paper-based records was highly labour-intensive and unsustainable for long-term studies.

Electronic recording of clinical data is now central to human and animal healthcare [92, 93]. Data collected from the 90% of UK veterinary practices that use electronic practice management systems (PMSs) can contribute enormously to clinical research [94–96]. The ‘Independent Inquiry into Dog Breeding’ report cited primary-care practice electronic clinical data using standardised coding of diagnoses as the optimal data source for reliable prevalence estimation [7]. However, early attempts at large-scale electronic surveillance struggled to cope with the large volumes of clinical data collected [19] and initial veterinary PMSs did not enforce structured coding systems [97], although there is now evidence that practising veterinarians accept a clinical rationale for standardised data recording [98, 99].

In the US, the National Companion Animal Study (NCAS) spanned 1992–1995 and analysed coded clinical data from 31,484 dogs treated at 52 first-opinion clinics [5]. Clinical diagnostic terms were recorded onto paper by attending clinicians before codification to a standardised nomenclature (PetTerms; developed dynamically during the study) and electronic transfer to a proprietary PMS [100].

The first of three published NCAS studies described age, breed, sex, diet and body condition score, and reported prevalence estimates for the most common disorders diagnosed [5]. However, the study was compromised by limiting the denominator population to just those animals with at least one coded diagnosis (36.3% of unique animal records), potential transcription error during the paper-to-electronic transfer of data and the absence of a prior-standardised coding system. Two further NCAS publications on obesity in cats [101] and dogs [102] demonstrated the potential to augment secondary EPR data with additional primary data collection on diet and body condition score for enhanced investigations [103]. The NCAS studies highlighted the importance to sustainable surveillance of standardised coding, direct recording of electronic data by clinicians, inclusion of all clinical care events and electronic integration between PMSs and research databases.

Eight hundred Banfield Pet Hospitals (http://www.banfield.com/) have generated clinical data on over 2.2 million dogs across 43 states in the USA [104]. Surveillance based on Banfield clinical data benefits from the use of a single PMS with daily uploads of standardised EPRs to a single computer server [105]. Collaborative studies using Banfield Pet Hospital data have reported on canine disorders including nematode parasitism, demodicosis, pancreatitis and atopic dermatitis (Table 2) while internal Banfield studies have been published online as ‘State of Pet Health’ reports [104].

**Table 2:** **Selected publications and findings on dog health based on Banfield Pet Hospital clinical data**

Topic	Study period	Reference
Gonadectomy, is a risk factor for obesity	1998-2010	Lefebvre *et al.* [106]
Vaccine-associated adverse events, multiple vaccines doses administered per visit increased risk	2002-2003	Moore *et al.* [107]
Tick infestation, younger, male and sexually intact dogs at increased risk	2002-2004	Raghavan *et al.* [108]
Periodontal disease, associated with cardiovascular-related conditions	2002-2006	Glickman *et al.* [109]
Periodontal disease, positive association with the incidence of azotaemic CKD	2002-2008	Glickman *et al.* [110]
Nematode parasitism, age, body weight, sex, breed and geographic region were risk factors	2003-2006	Mohamed *et al.* [111]
Tick infestation, systematic monitoring of veterinary and human medical data can improve detection in tick activity	2006-2007	Rhea *et al.* [103]
Pancreatitis, prevalence of 23 per 10,000 patients	2006	Lewis [112]
Demodicosis, American Staffordshire Terrier, Staffordshire Bull Terrier and Chinese Shar-pei at highest risk	2006	Plant *et al.* [105]
Atopic dermatitis, 1.7% prevalence	2007	Lund [113]
Castration, 64% prevalence	2007	Trevejo *et al.* [114]
Environmental monitoring, methods using veterinary records require further development	2006	Maciejewski [115]

The National Companion Animal Surveillance Program (NCASP) was developed at Purdue University in 2003 with a $1.2 million grant from the Centers for Disease Control and Prevention (CDC) to provide near real-time syndromic surveillance of pet animals as sentinels for bioterrorism, emerging zoonoses, toxic chemical exposures and for veterinary drug and vaccine pharmacovigilance [116]. Banfield EPR data were linked with Antech Diagnostics electronic laboratory reports from over 18,000 private veterinary practices [117]. Resultant publications on dog health have covered vaccine safety [19, 107, 118, 119], tick infestation [108] and toxic exposure [115]. However, NCASP surveillance was limited by confidentiality issues, delayed dissemination of results and difficulties in managing such large volumes of data [117]. It is reported that NCASP has been discontinued [120].

VetCompass (Veterinary Companion Animal Surveillance System) was developed at the Royal Veterinary College (RVC) in collaboration with the University of Sydney for companion animal surveillance using primary-care practice clinical data. A pilot phase, spanning 2007–2009, preceded implementation of the full UK project from September 2009 onwards. VetCompass holds clinical data on over 275,000 dogs from 189 UK practices (August 2013) (http://www.rvc.ac.uk/VetCompass/Index.cfm). Attending clinicians record VeNom code [22] summary diagnosis terms during episodes of clinical care [121]. Clinical data are automatically uploaded weekly to the VetCompass database [122]. Published VetCompass studies have covered pharmacotherapeutics [122], demography [123] and specific disorders [11, 121] of dogs. Current VetCompass projects aim to prioritise the welfare impact of common disorders in dogs, to evaluate the longitudinal course of canine mitral valve disease and to pilot the linkage of pedigree data to clinical health records in collaboration with the UK Kennel Club (KC) [47]. VetCompass has been developed in Australia and preliminary project work is underway in Spain, Germany and New Zealand. Realisation of the full surveillance potential of VetCompass has been constrained by limitations in automated information extraction from large datasets but current work to apply NLP methods offers promise [124].

The Small Animal Veterinary Surveillance Network (SAVSNET) was launched in 2008 at the University of Liverpool as a pilot project collecting data from UK veterinary diagnostic laboratories and veterinary practices [125] before becoming a registered charity in 2012 and entering a partnership with the British Small Animal Veterinary Association (BSAVA) [126]. A SAVSNET study of antibacterial prescribing patterns identified the importance of data validation for automated search strategies of primary-care practice data by showing substantial variation between the positive predictive value of four diagnoses, abscess (82%), diarrhoea (91%), cystitis (100%) and coughing (90%) [127]. A moderate negative impact from using opt-in consent was indicated by the 2.6% of clients who declined to participate [127], suggesting the relevance of appropriate consent protocols within project design [128]. Syndromic surveillance results are also posted on the project website [129].

The Centre for Evidence-based Veterinary Medicine (CEVM) was established in 2009 at the University of Nottingham [130]. The CEVM aims to promote the use of reliable and relevant science (or evidence) in clinical decision-making between veterinary surgeons and the owners of the animals. To facilitate this, the CEVM has created a small network of sentinel practices who they work very closely with, to look at the complexity of consultations, the reliability and limitations of EPRs and identify important areas for future research for veterinarians, owners and their animals.

To-date, primary-care practice EPR data have been an under-used surveillance resource [5]. Studies using EPR data may encounter misclassification problems [127] and technical difficulties related to management and analysis of large datasets [120]. Additionally, primary-care veterinary data mainly feature disorders that either prompted a veterinary-care visit or were detected during an otherwise veterinary examination and thus may miss that proportion of the overall disorder burden of dogs that does not receive veterinary attention. Just 44.1% of the true illness events in dogs are severe or persistent enough to lead to veterinary attention [131]. However, primary-care EPR research boasts increasingly large datasets for achieving good precision within study output [132] and the investigation of rare events [133], good prospects of generalisation to the wider dog population from the 70% of UK dogs that are registered with a veterinary practice [33, 46] and clinical relevance of the emergent results to practising veterinarians [127]. Cohort data collection can facilitate survival and co-morbidity analyses for chronic diseases [11, 134]. Ongoing developments in database management, analytic techniques, standardised coding and collaborative research design should enhance the surveillance role for primary-care practice EPR research [13].

### Veterinary cancer registries

Cancer registries systematically collect and analyse cancer data and are considered key to human cancer control, with 449 registries covering 21% of the world human population [135]. However, veterinary cancer registries are uncommon, often short-lived and suffer from poor communication and collaboration [136].

The VMDB began in 1964 as a hospital-based cancer registry [75, 137] and has published studies in dogs that covered cutaneous mast cell tumours [138], prostate carcinoma [139], cardiac tumours [82] and osteosarcoma [140]. Referral bias limits generalisability from VMDB study results to the wider dog population [12].

The Norwegian Canine Cancer Registry (NCCR) was established in 1990 and has reported results from studies that investigated mammary tumours in bitches [141, 142]. The Danish Veterinary Cancer Registry (DVCR) was established in 2005 as an online registry for passive veterinarian upload of clinical information [137]. Published DVCR studies have included cancer frequency in dogs in Denmark [143] and canine mast cell tumours [144]. The Animal Tumour Registry of Vicenza and Venice (Italy) was established in 2005 and used data on 2,509 samples submitted by 164 veterinary clinics to report cancer incidence in dogs. A denominator dog population was estimated using a telephone survey [145]. The Animal Tumour Registry of Genoa (Italy) used data from 6,743 canine tumour biopsies submitted between 1985 and 2002 to report cancer incidence in dogs. A denominator population was estimated using a capture-recapture methodology [146]. These studies acknowledged limitation from poor denominator population enumeration and used differing methods to estimate missing values. The telephone survey for population estimation benefitted from relative speed and additional collection of other useful demographic data.

Although useful for some risk factor studies, cancer registry clinical data are limited for disease surveillance in dogs by variable reporting, referral bias, denominator population enumeration problems and geographical variation [12, 147]. While under-reporting might generally be expected, cancer registries may also over-report certain neoplasias because of screening programs, research focus, new diagnostic modalities or free histopathology. Inconsistent inclusion criteria, nomenclature and classification schemes have limited comparisons of results across schemes. The use of standardised coding and diagnostic systems, data extraction directly from PMSs and cross-linking with pathology laboratory systems would enhance the application of veterinary cancer registry data for disease surveillance.

### Questionnaire-based data collection

Registered breeders represent a knowledgeable and important sub-population of dog-owners. The KC/BSAVA UK health survey of purebred dogs collected information from breeders on overall dog health, breeding and mortality as well as puppy birth defects. Of approximately 56,363 questionnaires forwarded to breed club members, 13,759 useable forms were returned (24%), representing 36,006 live dogs [148]. This large survey provided a useful resource of information on purebred dog health and mortality but generalisation was limited by the low response rate, absence of veterinary validation of reported disorders and inclusion of only those dogs owned by breed club members. Future studies aimed at breeder groups could benefit from linkage to veterinary diagnoses and greater prior involvement of breed clubs.

Information collected directly from the dog-owning public may improve representativeness of the overall dog population. The Pet Food Manufacturers Association (PFMA) 2012 survey used data from 2,159 face-to-face interviews to report demographic estimates for the UK dog population [149]. However, the lack of reporting on response rates, selection criteria, statistical weightings and confidence intervals limit the validity of the PFMA survey results. A UK general public telephone survey investigating dog ownership achieved a response rate 37% from a total of 1,656 calls and reported that 23.92% of households owned at least one dog [46]. A random-digit dialling telephone survey in Ireland generated 1,250 completed responses from 105,803 calls (1.2%) to describe dog and cat demography but was limited by the low completion rate [150].

Veterinary practice questionnaires can collect data from either the practice teams or their clients. A questionnaire circulated by email and post to 2,763 UK veterinary practices was used to report the number of practice-registered dogs but was limited by a low response rate (3.7%) [46]. A questionnaire distributed at UK veterinary clinics and dog shows investigating inter-dog aggression reported a completion rate of 3,897 from 14,566 distributed questionnaires (26.8%) [151]. An Australian study distributed questionnaires at veterinary clinics and pet shops to investigate owners’ attitudes towards obesity in dogs and reported a 36.5% response rate [152]. Integration of owner-recorded data offers the potential to complement veterinarian-derived data within broader study designs.

Questionnaire surveys are common methods for active collection of primary veterinary epidemiological data [153] and benefit from relative speed, repeatability, low cost and the capture of information on multiple risk factors and confounders. However, potential drawbacks associated with the use of questionnaires include low response rates, loss of information on temporality, difficulties with data validation, recall and non-responder bias, and ill-defined denominator populations [154]. Participation in questionnaire-based studies have been declining by about 1% annually from 1970 to 2003 because of generally decreased volunteerism, over-surveying, increasingly complicated surveys and conversion from landline to mobile phone use [155]. That said, increasing internet access has enhanced the promise for internet-based questionnaires [156] and careful study design can mitigate some of these drawbacks. Study designs that include nested questionnaires can benefit from focused primary data collection.

### Canine health schemes

Over 120 dog breeds have at least one DNA test available [157]. The results of formal DNA screening schemes co-ordinated by the KC are published online, providing surveillance data that can be linked with KC pedigree information on phenotype and parentage [158]. These data have been analysed to estimate the mutation prevalence for primary lens luxation among affected breeds [159]. However, selection bias arising from systematic avoidance of or intensive testing of known affected lines have limited generalisation from these studies to the wider dog population [160].

Brain stem auditory evoked response (BAER) testing distinguishes bilaterally and unilaterally hearing-impaired dogs from non-affected animals [161]. BAER data have been used to report prevalence estimates for deafness in Dalmatians [162], Border Collies [163] and Australian Cattle Dogs [164] as well as across multiple breeds [165]. These studies benefitted from well-defined case inclusion criteria and large study sizes but the voluntary submission process may have affected the presentation probabilities for known deaf individuals.

The BVA (British Veterinary Association)/KC hip dysplasia (HD) and elbow dysplasia (ED) schemes hold data on over 100,000 radiographs assessed since 1984. Dogs evaluated under these schemes must be permanently identified by microchip or tattoo [166] and breed median scores are published to assist breeding decisions [167]. Linkage between HD and ED schemes results and KC pedigree data has allowed pedigree evaluation and generation of estimated breeding values (EBVs) [168] for HD [169] and ED [170] in Labrador Retrievers. However, selection bias resulting from predominant inclusion of registered purebreds and the unlikelihood of submissions from dogs that are clinically affected or have obviously affected radiographs limit generalisation and may bias prevalence estimates downwards [171].

The BVA/KC/ISDS (International Sheepdog Society) eye scheme holds eye test data spanning over 30 years of testing for 11 hereditary eye conditions in over 50 breeds [172]. These data have been analysed to estimate the incidence [173] and inheritability [174] of multifocal retinal dysplasia in the Golden Retriever and the incidence of cataracts among Labrador Retrievers [175]. Eye scheme data for research are limited by misclassification bias from diagnosis difficulties for ocular disorders and selection bias from testing only a small proportion of KC-registered purebreds and very few non-registered dogs [173].

Canine health schemes generally harvest primary data from voluntary owner submissions and often benefit from well-characterised data, permanent animal identification (microchip, tattoo), openly published results and linkage with genetic databases [166]. However, intrinsic selection bias from passive collection processes and questionable representation of the overall dog population limit the generalisability of study results.

### Other companion animal surveillance systems in the UK

#### SARSS, Suspected Adverse Reaction Surveillance Scheme

Veterinary pharmacovigilance in the UK is monitored by the Veterinary Medicines Directorate (VMD) via SARSS using passive reporting of adverse events to veterinary medicines in both animals and humans [176]. The number of adverse events reported per year for dogs increased from 653 to 1,615 between the years 2003 to 2011 [177–185]. The 2011 result [182] equates to an average of only one report per year for every 10 of the 17,260 registered home-practising veterinary surgeons in the UK [95]. This suggests heavy under-reporting and likely selection bias with resultant questionable reliability of SARRS data for general surveillance purposes.

#### DACTARI, The Dog and Cat Travel and Risk Information

The DACTARI surveillance scheme was launched in 2003 by the Department for Environment Food and Rural Affairs with involvement from BVA and BSAVA as a national voluntary reporting scheme to monitor exotic diseases events in dogs and cats in Great Britain [186]. The scheme focuses on four disorders: leishmaniosis, babesiosis, ehrlichiosis and dirofilariasis. However, from 2001 to 2011, only 109 reports were received for these four diseases in dogs. Given that Bristol University reported 257 cases of canine leishmaniosis in the UK between 2005 and 2007 [187], this suggests heavy under-reporting to DACTARI of the true exotic disease burden.

#### CICADA, The Companion Animal Disease Survey

The CICADA survey is coordinated by MSD Animal Health (http://www.msd-animal-health.co.uk/) as a UK web-based disease surveillance scheme with voluntary online submissions of animal health data by veterinarians and professional organisations. The scheme aims to report disease trends, recent outbreaks and current hot spots [188]. Between October 2011 and June 2012, only 93 institutions (laboratories, universities or veterinary practices) submitted information to CICADA [189], suggesting high selection bias and limited generalisability to the wider animal population.

Rather than being useful for general surveillance, passive reporting systems may better suited for generating alarm signals that trigger more intensive targeted surveillance [19]. Passive collection may suffer from chronic under-reporting, inadequately defined denominator populations, selection bias and poor generalisation, and such systems are difficult to sustain in the long-term [190].

## Conclusions

Increasing demand for veterinary surveillance is constrained by decreasing availability of human and financial resources [191]. Credible canine health surveillance requires a reliable data source with a well-defined denominator population, evidence of representativeness and validity and appropriate study design [4]. Active data collection is preferred for veterinary surveillance because of reduced selection bias and known selection probability for each epidemiological unit that permit estimation of absolute, rather than relative, risk values [2]. Secondary data are becoming increasingly important for companion animal surveillance because of their collection efficiency, reduced costs, larger sample size, better representativeness and reduced bias [14]. Integration between data sources can facilitate knowledge extraction and interpretability within individual studies and underlines the epidemiological importance of permanent identification of individual animals [192, 193].

Many data sources have been recruited for canine health surveillance, each with distinct prevailing advantages and limitations (Table 3). Insurance data benefit from large and well-defined denominator populations but are limited by selection bias relating to the clinical events claimed and animals covered. Veterinary referral clinical data offer good reliability for diagnoses but are limited by referral bias for the disorders and animals referred. Primary-care practice EPR data benefit from strong alignment with the general dog population and veterinary validation but encounter technical difficulties related to the management and analysis of large datasets. Veterinary cancer registries offer good diagnostic reliability but may have ill-defined denominator populations and poor representativeness. Questionnaire surveys are relatively inexpensive and collect primary data but suffer from issues relating to validation and response rates. Canine health scheme data benefit from well-characterised disorder and animal data but are subject to selection bias from the voluntary submission process. Formal UK passive surveillance systems are limited by chronic under-reporting and selection bias. It is concluded that active collection systems using secondary health data currently provide the optimal single resource for canine health surveillance and that linking multiple data sources can substantially amplify the research potential.

**Table 3:** **Advantages and limitations to data sources used for health surveillance in dogs**

Data source	Advantages	Limitations	Applications
Pet insurance databases	Large size	Difficult to validate	Agria Pet Insurance data analysis in Sweden [15]
	Defined denominator	Questionable representativeness of the general population	Pet Protect insurance data analysis in UK [58, 69]
	High reliability for breed and sex	Loss of data on low-cost or excluded disorders	
	Coded diagnoses		
Referral practice clinical records	Good diagnostic reliability?	Referral bias	Veterinary Medical Data Base (VMDB) [75]
	Coded diagnoses?	Poorly defined denominator	
	Large databases	Poorly representative	
Primary-care practice clinical records	Large databases	Diagnostic reliability?	Banfield Pet Hospital [104]
	Highly representative?	Technical complexities	NCAS [5]
	Coded diagnoses	Only events with veterinary care	NCASP [116]
	Defined denominator		VetCompass [47]
	Generalisability		SAVSNET [129]
			CEVM [130]
Veterinary cancer registries	Human registries common	Referral bias	Veterinary Medical Data Base (VMDB) [75]
	Good diagnostic reliability	Poorly defined denominator	Danish Veterinary Cancer Registry [137].
		Poorly representative	
Questionnaire-based data collection	Relatively inexpensive	Response rate	The KC/BSAVA UK health survey of purebred dogs [148].
	Flexible	Difficult to validate	
	Can nest within other study designs	Loss of information on temporality	
Canine health schemes	Large databases	Poorly representative	BVA/KC hip dysplasia and elbow dysplasia scheme [194]
	Diagnostic reliability	Selection bias	BVA/KC elbow dysplasia scheme [195]
	Linkage to KC pedigree data		The BVA/KC/ISDS eye scheme [172].
	Permanent animal identification		
Other companion animal surveillance systems in the UK	Relatively inexpensive	Under-reporting	SARSS [176]
		Poorly defined denominator	
		Selection bias	DACTARI [186]
		Poor generalisability	CICADA [189]

## Abbreviations

AAHA: American Animal Hospital Association
BSAVA: British Small Animal Veterinary Association
BVA: British Veterinary Association
CDC: Centers for Disease Control and Prevention
CEVM: The Centre for Evidence-based Veterinary Medicine
CICADA: Companion Animal Disease Survey
DACTARI: Dog and Cat Travel and Risk Information
DVCR: Danish Veterinary Cancer Registry
EBV: Estimated breeding value
ED: Elbow dysplasia
EPR: Electronic patient record
HD: Hip dysplasia
ISDS: International Sheepdog Society
KC: The Kennel Club
NCAS: National Companion Animal Study
NCASP: National Companion Animal Surveillance Program
NCCR: Norwegian Canine Cancer Registry
PFMA: Pet Food Manufacturers Association
PMS: Practice management systems
RVC: Royal Veterinary College
SARSS: Suspected Adverse Reaction Surveillance Scheme
SAVSNET: Small Animal Veterinary Surveillance Network
VetCompass: Veterinary Companion Animal Surveillance System
VMD: Veterinary Medicines Directorate
VMDB: Veterinary Medical Database.
